# Trash Talking: Anthropogenic Resources Facilitate Raccoon Interactions in Urban Environments

**DOI:** 10.1002/ece3.72559

**Published:** 2025-12-08

**Authors:** Laura Dudley Plimpton, Meredith VanAcker, Sara Kross, Ximena A. Olarte‐Castillo, Sara Evans, Christopher R. Konowal, Meggan Craft, Laura B. Goodman, Gary Whittaker, David Needle, Maria Diuk‐Wasser

**Affiliations:** ^1^ Department of Ecology, Evolution, and Environmental Biology Columbia University New York New York USA; ^2^ College of Natural and Agricultural Sciences University of California, Riverside Riverside California USA; ^3^ School of Biological Sciences University of Canterbury Christchurch New Zealand; ^4^ Department of Microbiology & Immunology and James A. Baker Institute for Animal Health, College of Veterinary Medicine Cornell University Ithaca New York USA; ^5^ Department of Horticulture The Green‐Wood Cemetery Brooklyn New York USA; ^6^ Department of Ecology, Evolution, and Behavior University of Minnesota St Paul New York USA; ^7^ Department of Public and Ecosystem Health and James A. Baker Institute for Animal Health College of Veterinary Medicine, Cornell University Ithaca New York USA; ^8^ Department of Microbiology and Immunology and Department of Public and Ecosystem Health College of Veterinary Medicine, Cornell University Ithaca New York USA; ^9^ New Hampshire Veterinary Diagnostic Laboratory College of Life Sciences and Agriculture, University of New Hampshire Durham New York USA

**Keywords:** animal movement, anthropogenic resources, contact, intraspecific interaction, raccoon, resource patterning, social behavior, urban ecology

## Abstract

Interactions between animals of the same species underpin many ecological processes, from reproduction to pathogen transmission. Habitat modification, such as urbanization, affects an animal's spatial behavior, altering interactions with both their habitat and conspecifics. Raccoon space use varies widely between urban/suburban and rural populations, with anthropogenic resources suggested as a key factor in shaping movement behavior and consequently, opportunities for conspecific interaction. Here, we use high‐resolution GPS data to identify instances of close spatiotemporal proximity (i.e., co‐occurrence), referred to as “contacts,” among raccoons in an urban greenspace in Brooklyn, New York City (NYC). To understand how resource patterning affects contact formation processes and evaluate possible spatial and demographic factors contributing to the types of contact observed, we evaluated the effect of proximity to different resources (including anthropogenic subsidies) on the probability of urban raccoon contact and assessed associations between the characteristics of urban raccoon contact events. We found that certain resources increase the likelihood of urban raccoons coming into contact, with the largest positive effect observed for anthropogenic resources. Shared characteristics across contact events suggest three main types of co‐occurrence: (1) longer duration contacts between males near anthropogenic resources, (2) proximity between females near fruiting plants or while denning, and (3) transient interactions between males and females. We conclude that in an urban habitat, anthropogenic subsidies are important drivers of co‐occurrence between raccoons, which interact dynamically with social factors to shape the characteristics, frequency, and distribution of contacts across the urban landscape. Our data have important implications for predicting the dynamics of contact‐driven processes—particularly pathogen transmission—in urban raccoon populations.

## Graphical abstract

Interactions between conspecifics play a key role in ecological processes, and habitat modification, such as urbanization, can alter the spatial and social behaviors that underpin such interactions. Here, we used high‐resolution GPS data to examine how resource distribution affects raccoon contact in an urban greenspace in Brooklyn, NYC. We conclude that anthropogenic subsidies are important drivers of interaction between urban raccoons, which interact dynamically with social factors to shape the frequency and distribution of contacts across the landscape, with implications for the management of urban wildlife.

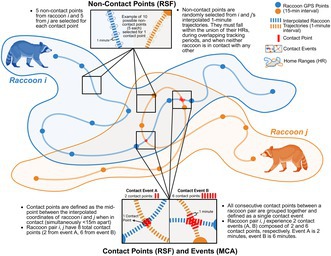

## 1 Introduction

Anthropogenic landscape change alters not only where animals move but also how they interact (Hoover and Tylianakis 2012; Doherty et al. 2021). Intraspecific interactions are shaped by spatial and behavioral processes that together govern where individuals are located over space and time and how they respond to conspecifics (Whitehead 2008; Webber et al. 2023; Gaynor et al. 2024). Resource patterning, in particular, influences both movement and social behavior and therefore plays a central role in facilitating intraspecific interactions (Kappeler et al. 2013; He et al. 2019). Shifts in resource distribution, often associated with anthropogenic landscape change, can modify the spatial structure of wildlife populations and thus, the frequency or nature of interactions, which underpin various ecological processes from reproduction to pathogen transmission (Fahrig 2007; Sih et al. 2010; Hoover and Tylianakis 2012; Gottdenker et al. 2014; Becker et al. 2015, 2018; Doherty et al. 2015; Wong and Candolin 2015).

Urban areas represent some of the most heavily modified landscapes, particularly in terms of resource availability and distribution. A defining characteristic of cities is the abundance of municipal waste, which offers a novel, predictable, and often aggregated food resource to urban wildlife. These resources can reduce energetic costs associated with foraging, increase caloric intake, and buffer against seasonal or stochastic resource scarcity (Oro et al. 2013; Ruffino et al. 2014). As a result, anthropogenic subsidies can elevate local carrying capacities, inflate population densities, and shift age structures or reproductive timing (Prange et al. 2003; Nagy and Holmes 2005; Oro et al. 2013). Such demographic outcomes are often accompanied by movement changes, including smaller home range sizes, increased site fidelity, and reduced but concentrated foraging efforts (Prange et al. 2004; Bino et al. 2010; Wehtje and Gompper 2011; Santonastaso et al. 2012; Oro et al. 2013; West et al. 2016). Together, these shifts promote increased spatial overlap among conspecifics, particularly around high‐value resource patches (Wehtje and Gompper 2011). In such contexts, selective tolerance and even group formation may emerge if the benefits of co‐using these patches outweigh the costs of competition. For example, the Resource Dispersion Hypothesis (RDH) proposes that aggregated, high‐quality resources reduce the costs of proximity and facilitate passive aggregation among species that are typically solitary (Carr and Macdonald 1986; Johnson et al. 2002; Macdonald and Johnson 2015). As a result, even otherwise solitary urban wildlife may exhibit increased tolerance, creating opportunities for contact through shared use of clustered resources.

Raccoons ( *Procyon lotor* ) are behaviorally flexible, omnivorous mesomammals that thrive in cities and exhibit marked shifts in space use and conspecific tolerance in response to urban anthropogenic subsidies (Pedlar et al. 1997; Prange et al. 2003, 2004). For example, raccoons shift their home ranges in response to the location of clumped food sources and often maintain smaller ranges with greater spatial overlap in urban areas (Wehtje and Gompper 2011; Schuttler et al. 2015). While usually solitary (Fritzell 1978; Chamberlain and Leopold 2002), urban raccoons exhibit increased conspecific tolerance and even sociality; in particular, male raccoons are known to form small groups where population densities are high as a means of ensuring mate and resource access (Gehrt and Fritzell 1998b; Gehrt et al. 2008; Prange et al. 2011; Hirsch et al. 2013a; Reynolds et al. 2015). These behavioral changes restructure the frequency and context of intraspecific interactions with direct implications for the dynamics of contact‐mediated processes, like pathogen transmission.

Raccoons are hosts to a range of pathogens, some of which are of significant concern for human and domestic animal health, including rabies virus, Canine Distemper Virus (CDV), and *Leptospira* sp. Increased density, spatial overlap, and conspecific tolerance around shared resource patches increase contact opportunities, facilitating the transmission of pathogens through proximal encounters or closely timed use of shared resources (Hirsch et al. 2013b; Becker and Hall 2014; Becker et al. 2015, 2018). For example, anthropogenic subsidies have been associated with a higher prevalence of endoparasites in raccoons like *Bayliscaris procyonis*, a roundworm that can cause severe disease and death in humans, and increased opportunities for both intra and interspecies transmission (Totton et al. 2002; Wright and Gompper 2005; Wehtje and Gompper 2011). Therefore, understanding the factors that create opportunities for intraspecific interactions—and, in turn, pathogen exposure and transmission—is crucial in urban environments where raccoons and people live at high densities and in close proximity.

Here, we used high‐resolution GPS tracking to identify opportunities for close spatiotemporal proximity, hereafter referred to as “contacts,” among an urban raccoon population inhabiting a large cemetery and arboretum in Brooklyn, New York City (NYC). We then evaluated how different resources, including anthropogenic subsidies, influenced opportunities for contact among raccoons while accounting for underlying resource use, thus assessing if contact occurred near certain resources more often than would be expected given solitary use of each feature (Houston and McNamara 1988; Prugh et al. 2019; Albery et al. 2021; Yang, Boughton, et al. 2023). While we did not observe or validate the behavioral outcome of these events (e.g., aggressive, affiliative), these instances of possible contact still carry particular relevance to our system. This focal population experienced outbreaks of Canine Distemper Virus (CDV)—a highly infectious morbillivirus spread via aerosols, direct interaction, and contact with bodily secretions—in 2020, 2022, and 2023, making disease control a priority for local wildlife management. Given this concern, we define contact in our system to include even brief, non‐affiliative co‐occurrences or inferred proximity via closely timed use of shared resources, as such encounters may be sufficient to facilitate spread, and interpret our findings in the context of pathogen transmission. We hypothesized that (1) raccoon contact would be significantly associated with anthropogenic resources, even after accounting for individuals' independent use of those resources, reflecting increased tolerance consistent with RDH, and that (2) the characteristics of raccoon contacts would align with prior observations of increased tolerance and emerging sociality in urban raccoons, particularly among males.

## 2 Materials and Methods

### 2.1 Study Site

Our study site is a 193‐ha cemetery and arboretum, The Green‐Wood Cemetery, located in south Brooklyn, NYC. The cemetery is characterized by variable topography shaped by glacial moraine, with rolling hills and valleys as well as several kettle ponds and fabricated detention ponds. The neighborhood surrounding Green‐Wood is highly urbanized, comprising of connected residential brownstones/row houses with small backyards, mixed‐use low‐rise apartment buildings with shops/restaurants on the ground floors, and industrial lots/warehouses. Despite being embedded in the urban matrix, the cemetery and its neighboring parks are home to a large population of raccoons, which often cause conflict with residents and experience disease outbreaks (notably, CDV in 2020, 2022, and 2023).

### 2.2 Movement Data

Between August and late September 2022, raccoons, skunks ( *Mephitis mephitis* ), opossums ( *Didelphis virginiana* ), and free‐ranging, unowned cats ( *Felis catus* ) were live trapped across the cemetery and in neighboring residential backyards using Tomahawk traps (30.5 cm height × 33.0 cm width × 81.3 cm length, Tomahawk live trap, Hazelhurst, WI, USA) baited with cat food. Each animal was sedated using ketamine‐dexmedetomidine administered by a veterinarian (dosages adjusted for species and individual body weight) prior to a brief physical exam and sample collection. Samples collected included nasal/saliva/rectal swabs, whole blood and serum samples, a tissue biopsy from the pinna, and ectoparasites including ticks and fleas. Raccoons were tagged with small (~5 cm) numeric ear tags to identify individuals upon recapture (e.g., RA‐223). A subset of adult raccoons captured in the western ~60–70‐ha area of the cemetery (termed ‘collaring zone’) were fitted with GPS tracking collars weighing less than 8% of the animal's body weight (Smart Parks, Netherlands) (IACUC Protocol #: AC‐AABS3611; NYSDEC License #: 2704). To detect contact opportunities among a population of our estimated size (60–70 raccoons across the collaring zone), 32%–40% of the population should be tracked (Gilbertson et al. 2021). Thus, we aimed to fit 19–24 raccoons, evenly distributed across sexes, with GPS collars. Collars were sized to each animal and connected with a cotton spacer designed to degrade over the collaring period (3–6 months). The GPS collars were programmed to record the animal's position every 15 min between 7 pm and 7 am with additional daytime locations recorded every 4 h. GPS data was logged on internal storage as well as sent live over a locally constructed LoRaWAN network consisting of three gateways positioned across the cemetery. Complex topography can sometimes impede the connection between data packages and the receiving gateway, so whenever possible (e.g., upon recapture) data was downloaded directly from the internal collar storage.

### 2.3 Movement Models and Home Range Estimation

Initial filtering of GPS points included removing all 2D points with position dilution of precision (pDOP) values greater than five, as well as any locations with less than three satellites in view. Relocation data from recaptured raccoons was removed from downstream analyses starting at 8 pm on the recapture night until 2 pm the following afternoon to eliminate biased movements associated with trapping. We verified range residence, which describes the tendency of individuals to remain in a defined region, for each raccoon by plotting the estimated semi‐variance of distance between locations as a function of a time lag using the continuous‐time movement model package, *ctmm* in R (Calabrese et al. 2016). Individuals whose variograms reached an asymptote, indicating that the individual's relocation data captures the area of the movement process, were labeled as range residents (Fleming et al. 2014). Only individuals that were range residents and had a minimum of 150 relocations were included in downstream analyses. We assessed speed and distance outliers in the relocation data for each individual using the outlier function in *ctmm*. Continuoustime movement models (CTMM) were fit to each individual's relocation data using the ctmm.select function, which uses a starting prototype model with estimated parameters derived from the variogram for each individual and then identifies the best fit CTMM via the perturbative hybrid residual maximum likelihood (pHREML) method. Model selection was based on Akaike's Information Criterion adjusted for small sample sizes (AICc). The home range (HR) during late summer/early fall for each individual was then estimated using a weighted, area‐corrected, autocorrelation kernel density estimation (wAKDEc) conditional on the selected movement model for each individual in the *ctmm* package. Average home range crossing time was estimated from the best fit autocorrelation model for the population to validate if the monitoring period for each individual was long enough to capture multiple traversals of the HR (Fleming et al. 2019).

### 2.4 Contact Points

**FIGURE 1:**
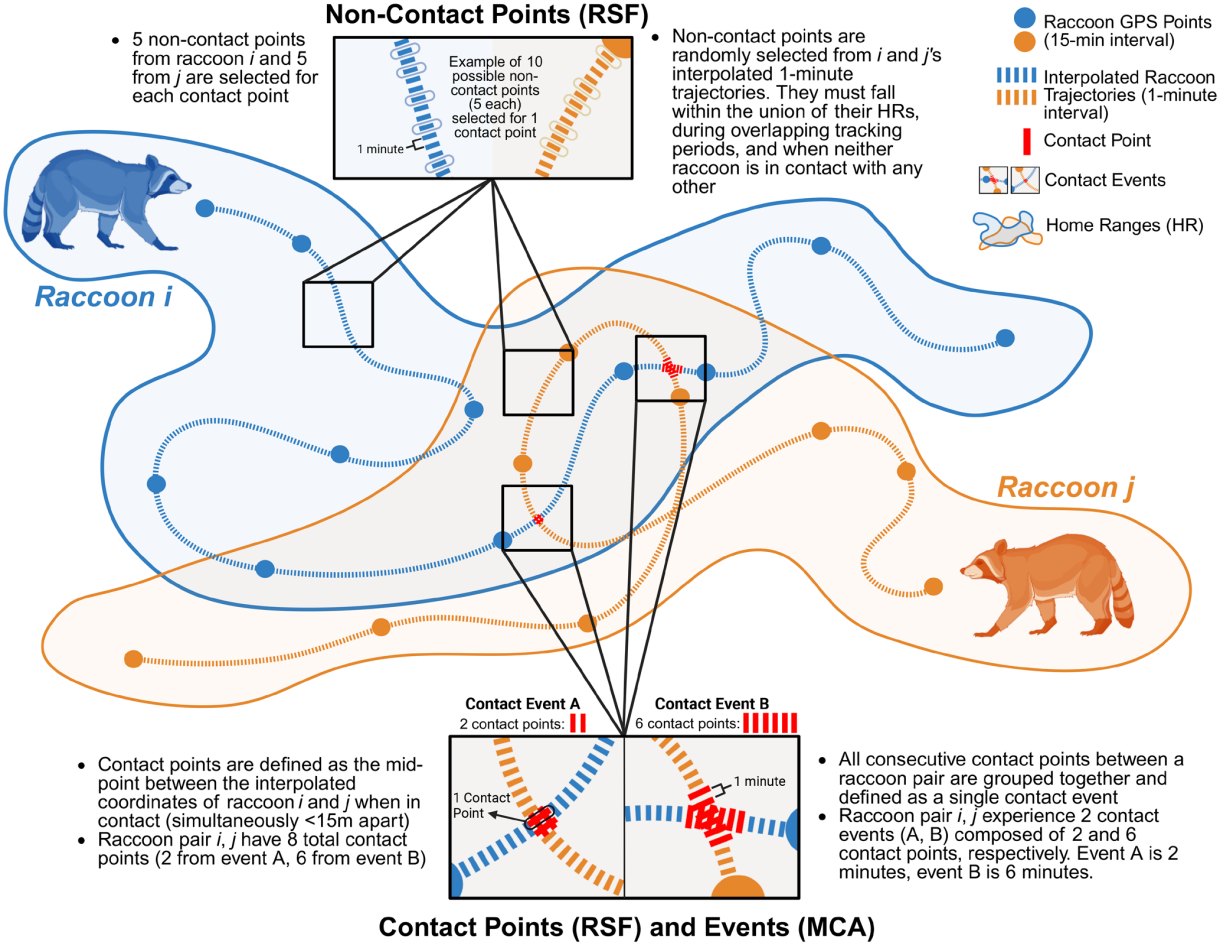
Schematic of raccoon contact analysis. Possible locations of contact (referred to as “contact points”) were identified along each raccoon dyad's interpolated trajectories and defined as co‐location at the same time within 15 m of each other. Contact points were used in the contact RSF analyses. For contact typology (MCA) analyses, consecutive contact points (i.e., time lag between points ≤ 1 min) for a dyad were grouped together and redefined as a contact event. A detailed schematic of the analytical pipeline is provided in Figure S1.

We adapted a method developed by Yang, Wilber, et al. 2023 to identify locations of spatiotemporal proximity, termed here “contact points,” which represent the opportunity for contact between raccoons. Briefly, this method fits a CTMM to the observed GPS data for each individual using the ctmm.guess and ctmm.select functions in the *ctmm* package. Next, the parameterized CTMM for each individual is used to predict its movement trajectory at a finer temporal resolution than the observed GPS fixes. Trajectories from every possible pairing of raccoons (i.e., dyad) are then overlaid to identify locations of spatiotemporal overlap termed contact points, which are defined as the co‐location of two individuals within a predetermined temporal window and spatial distance, informed by the GPS error. Given the concerns over pathogen transmission in this population—in particular CDV, which is highly infectious and transmitted through direct contact or aerosols—we interpolated each raccoon's trajectory to a 1‐min temporal resolution and defined contact points (i.e., opportunities for interaction) as all points along a raccoon dyad's trajectories where they are 15 m or less from each other (Figure 1). The midpoint between individuals when in spatiotemporal proximity (i.e., < 15 m) was then used to represent the location of contact for the dyad (i.e., contact point). This 15 m threshold accounts for typical GPS error and defines a conservative window within which raccoons may detect and initiate interaction with one another (Kent and Tang‐Martínez 2014; Morton 2021; Buzuleciu et al. 2016). While this does not confirm direct contact, it captures opportunities for interaction that form the basis of contact‐mediated processes and aligns with prior interpolated movement studies using similar proximity‐based criteria (e.g., Williams et al. 2014; Wilber et al. 2022; Yang, Wilber, et al. 2023; Egan et al. 2024). To increase confidence that contact points represented meaningful interaction opportunities for a dyad, we retained only those consisting of at least two consecutive 1‐min co‐occurrences.

### 2.5 Contact Resource Selection Function (RSF)

We utilized a resource selection function (RSF) modeling framework to evaluate the probability of raccoons coming into contact near certain resource features given the underlying use of each resource by each raccoon unrelated to contact. This method, which is an adaptation of the method described by Yang, Boughton, et al. 2023, applies a similar ‘used‐available’ framework to classic RSFs but instead defines the location of all possible contacts for each dyad as used points (see Contact Points). The available points are non‐contact points, which we define as locations along a raccoon's interpolated 1‐min trajectory where they are not within 25 m of any other raccoon (1.5× the contact distance threshold). For each contact point, 10 non‐contact points are selected at random without replacement from each individual's 1‐min trajectories (five from each individual in the dyad). Non‐contact points must be within the union of the dyad's home range (95% wAKDEc) and occur during their overlapping tracking period when neither individual is in contact with each other or any other collared raccoon (Figure 1).

We identified five resources across our site that we hypothesized influence urban raccoon space use and interactions: anthropogenic resources (e.g., residential trash, cat feeding stations), fruiting plants, denning trees, meadows, and water bodies. Within Green‐Wood, trash receptacles were the primary anthropogenic resource and were identified by walking the landscape and mapping point locations of trash cans. Due to the abundance of non‐visible anthropogenic resources in the adjacent urban area, such as backyard composting or cat feeding, we categorized anthropogenic resources outside the cemetery based on building type/use (NYC MapPLUTO) and assumed accessibility to raccoons. One‐and‐two‐family homes, multi‐family walk‐up buildings, mixed residential and commercial buildings, and public facilities and institutions all consistently kept trash outdoors either in cans or dumpsters. Most one‐and‐two‐family homes and some multi‐family walk‐up buildings also had backyard areas, which may provide anthropogenic resources for raccoons (e.g., barbeques, cat colonies). For each of these building types, we excluded the interior footprint and classified a 2 m buffer around each building's exterior as an anthropogenic resource. Green‐Wood maintains a plant record database of all woody plants including location, species, and size (diameter at breast height, DBH). Fruit/berry‐producing resources were classified as the point locations of all plants that produce fruits/berries during the mid‐summer to early fall (i.e., July–October; overlapping with our study period) in NYC. Denning trees were classified as the point locations of all trees with a DBH > 76.2 cm, which provide large enough branches or cavities for raccoons to den (Endres and Smith 1993; Smith and Endres 2012; Owen et al. 2015). In the proximate urban environment, potential denning trees were identified from Ma et al. (2023)'s NYC tree database and defined as trees with a volume > 1000. Green‐Wood provided polygon shapefiles of established wildflower meadows and all water bodies within the cemetery. A 2 m buffer was applied around all point locations of resources (e.g., trash cans, den tree locations, fruiting plant locations) before calculating the distance from every 0.25 m x 0.25 m cell to the nearest feature for each resource type. We then extracted the distance from all used (contact) and available (non‐contact) points to each resource type and tested all distance variables for multicollinearity (Pearson's correlation coefficient |*r*| ≥ 0.6) before centering and scaling.

To generate a population level contact‐RSF function, we implemented a generalized linear mixed effects model with a weighted logistic regression, binomial error distribution, and logit‐link function using the R package *glmmTMB* (Brooks et al. 2017). To account for differences in overlapping tracking periods for each dyad and dyad‐specific variation in availability and response to difference resources, we included random intercepts for each dyad, fixed the variance of the intercept at a large value (1,000,000), and included random slopes for all explanatory variables for each dyad (Muff et al. 2020). To meet assumptions of an inhomogeneous point process model, we assigned a weight of 1000 to available (non‐contact) locations and 1 to used (contact) locations. To ensure reliable statistical inference and address singularity issues, we only included data from raccoon dyads with at least 10 contact points representing at least two distinct instances of co‐occurrence, termed “contact events” (see below, Contact Typologies) in the models. We tested different combinations of the five resource features and selected the most parsimonious model based on AIC. To validate the final model, we regressed the observed against predicted data (Johnson et al. 2006; Bühler et al. 2023; Table S4). To assess model results, we extracted population level β coefficients using the extract_fixed_effects function and dyad‐level random coefficients using the extract_random_coefficient function both in the R package, *mixedup* (Clark 2022). The exponentiated value of the β coefficients is referred to as the relative selection strength (RSS). To evaluate agreement between population‐level RSS for each resource and dyad‐level coefficient estimates, we exponentiated both the population and dyad‐level coefficients and then plotted the population‐level RSS for each resource and the 95% confidence interval alongside the exponentiated random coefficients for each dyad and resource (i.e., random slopes) using the R package *ggplot2* (Wickham 2016).

### 2.6 Contact Typologies

Discrete, continuous instances of prolonged spatiotemporal proximity, hereafter termed “contact events,” were identified by grouping together all consecutive contact points (i.e., 1‐min lag) between each raccoon dyad (Figure 1). For each contact event, we selected the point during which the pair was at the shortest distance apart to represent the event's location. We summarized each resource type (denning tree, anthropogenic, fruiting plant) as a binary layer in which the area within a 15 m buffer of the resource was classified as 1 and all grid cells not associated with that resource type were classified as 0. All resource layers were rasterized to a 0.25 m × 0.25 m grid. We then extracted the binary classification of each resource type for each contact event's location. The duration of each event was summarized based on quartiles in the data: short duration (1–3 min), medium duration (4–6 min), and long duration (> 7 min). Last, we recorded the sexes of the raccoon dyad involved in each event (male–male, female–female, male–female) and the time of day that the event occurred (foraging hours, 9:30 pm–4:30 am vs. denning hours, 7 pm–9:30 pm/4:30 am–7 am).

To identify associations between categories of the different variables (duration, sex pairing, time of day, resource category) describing the contact events, we conducted a multiple correspondence analysis (MCA) using the R package FactoMiner (Lê et al. 2008). The correlation between the variables and MCA principal dimensions was visualized using the function fviz_mca_var in FactoMiner. Additionally, the squared cosine distance (cos2), which indicates the confidence in the placement of each variable category on the axes, and the contribution of each variable category to each dimension were both visualized. For each variable, we colored the events by the respective category and added an ellipse around each variable category using the function fviz_mca_ind in FactoMiner. These visuals were used to qualitatively characterize different types of contact events (i.e., contact typologies) observed among the raccoons and assess associations between spatiotemporal proximity and raccoon natural history and space use.

## 3 Results

### 3.1 Movement Summary

Over 14 nights between August and mid‐September 2022, we captured 37 raccoons in/around Green‐Wood Cemetery. Of these, 32 raccoons (17 males and 15 females) were located within the collaring zone.

Because some raccoons were too young and did not meet our weight requirements (> 3 kgs), only 24 of the 32 raccoons were fitted with collars (Figure S2). Due to collar failures and raccoons wearing out the cotton spacers more quickly than anticipated, locational data were recovered from 22 of the 24 individuals. Of the 22 individuals, 19 (9 males and 10 females) had adequate amounts of data and were range residents, and so were included in downstream contact probability (contact RSF) and contact typology (MCA) analyses.

### 3.2 Raccoon Home Range Analyses

**FIGURE 2:**
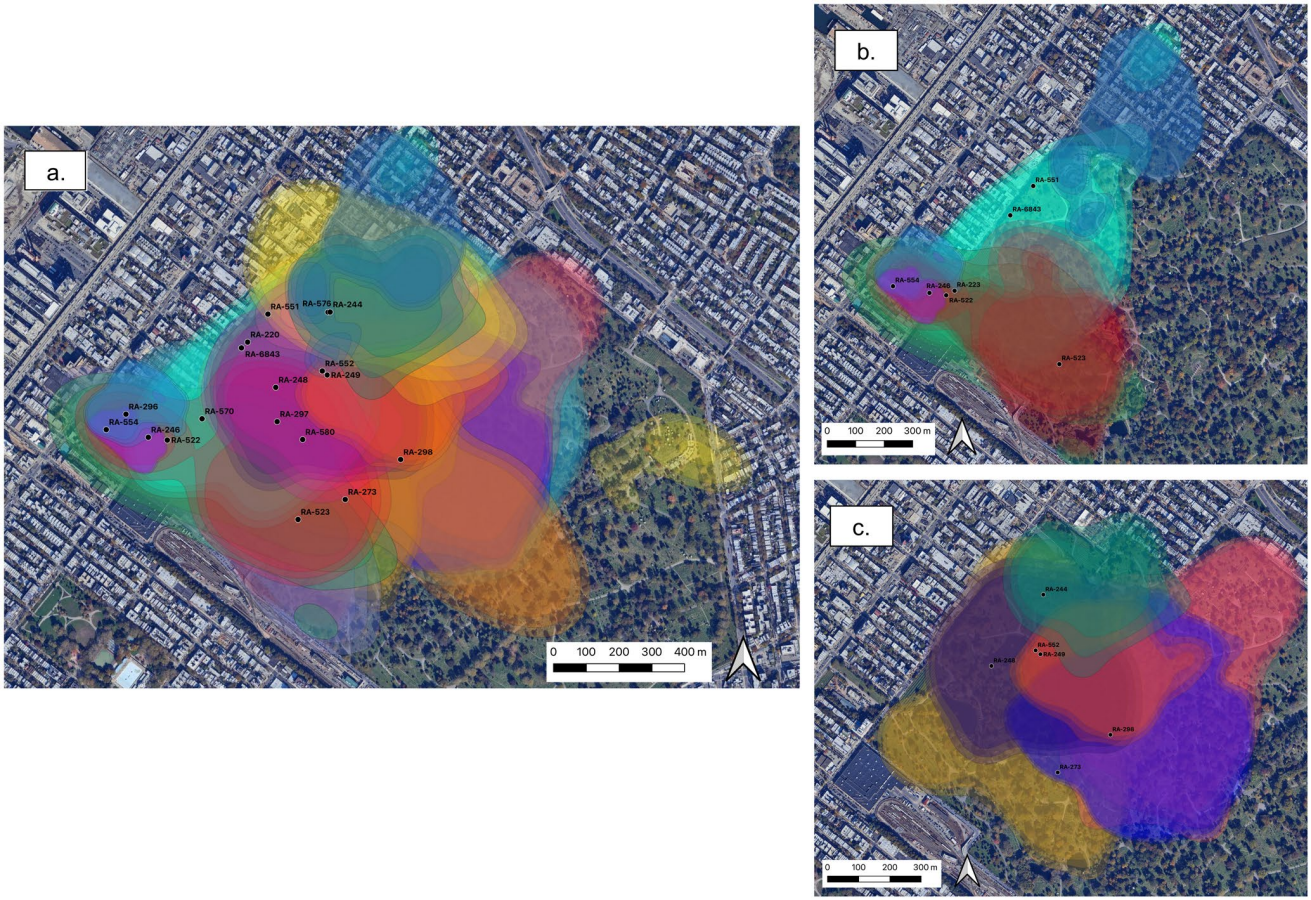
Home ranges of Brooklyn raccoons. Middle contours represent the area where the animal spends 95% of its time during the late summer/early fall (wAKDEc) and the outer and inner contours correspond to the upper and lower confidence intervals, respectively. Black points show the initial capture locations of each raccoon. Polygons in cool colors (purple/blue/green) are males and polygons in warm colors (yellow/orange/red/pinks) are females. (a) wAKDEcs of all 19 raccoons (b) wAKDEcs of urban/urban edge raccoons (c) wAKDEcs of cemetery‐based raccoons.

Individual weighted autocorrelated kernel density (wAKDEc) home range estimates based on the best fit continuous‐time movement model for each individual were derived for 19 of the raccoons (Figure 2). Monitoring time ranged from a minimum of 4.96 days (RA‐244) to 88.6 days (RA‐273) (Table S1). Individual 95% wAKDEcs ranged in size from 2.83 ha (RA‐246) to 76.08 ha (RA‐570) with a mean of 32.87 ha (Figure S3). Individuals with the smallest home ranges (e.g., RA‐246, 2.83 ha; RA‐554, 5.18 ha) foraged along the edges of the cemetery or within the urban matrix (Figure 2b), while individuals with the largest home ranges remained primarily in the cemetery (Figure 2c). Raccoons that foraged within Green‐Wood generally remained within the boundary of the cemetery with the exception of the service yard, an area in the southwest corner of Green‐Wood that contains burial space as well as utility services and operational equipment. The service yard sits outside of the main boundaries of the cemetery but is directly accessible through a viaduct that crosses under 5th Avenue, a 4‐lane road with traffic in both directions—which may have acted as a permeable edge allowing primarily cemetery‐based raccoons (e.g., RA‐223, RA‐296, RA‐523) access to the bordering anthropogenic environment. Raccoons that lived outside of the cemetery occasionally visited Green‐Wood and when they did, only utilized a small area confined to the edges or the service yard (e.g., RA‐ 246, RA‐554, RA‐551). Average home range crossing time estimated from the best fit autocorrelation model for the population indicated that, on average, raccoons cross their home range in 2.81 h. Based on the best fit individual‐based autocorrelation models, the maximum estimated time it took for an individual to cross its home range was 8.55 h (RA‐223) and the minimum time was 1.06 h (RA‐246) (Table S1). Thus, a minimum monitoring time of ~5 days is likely to capture movement and interactions across each individual's late summer/early fall home range that is representative of behavior at this time.

### 3.3 Contact Points

**FIGURE 3:**
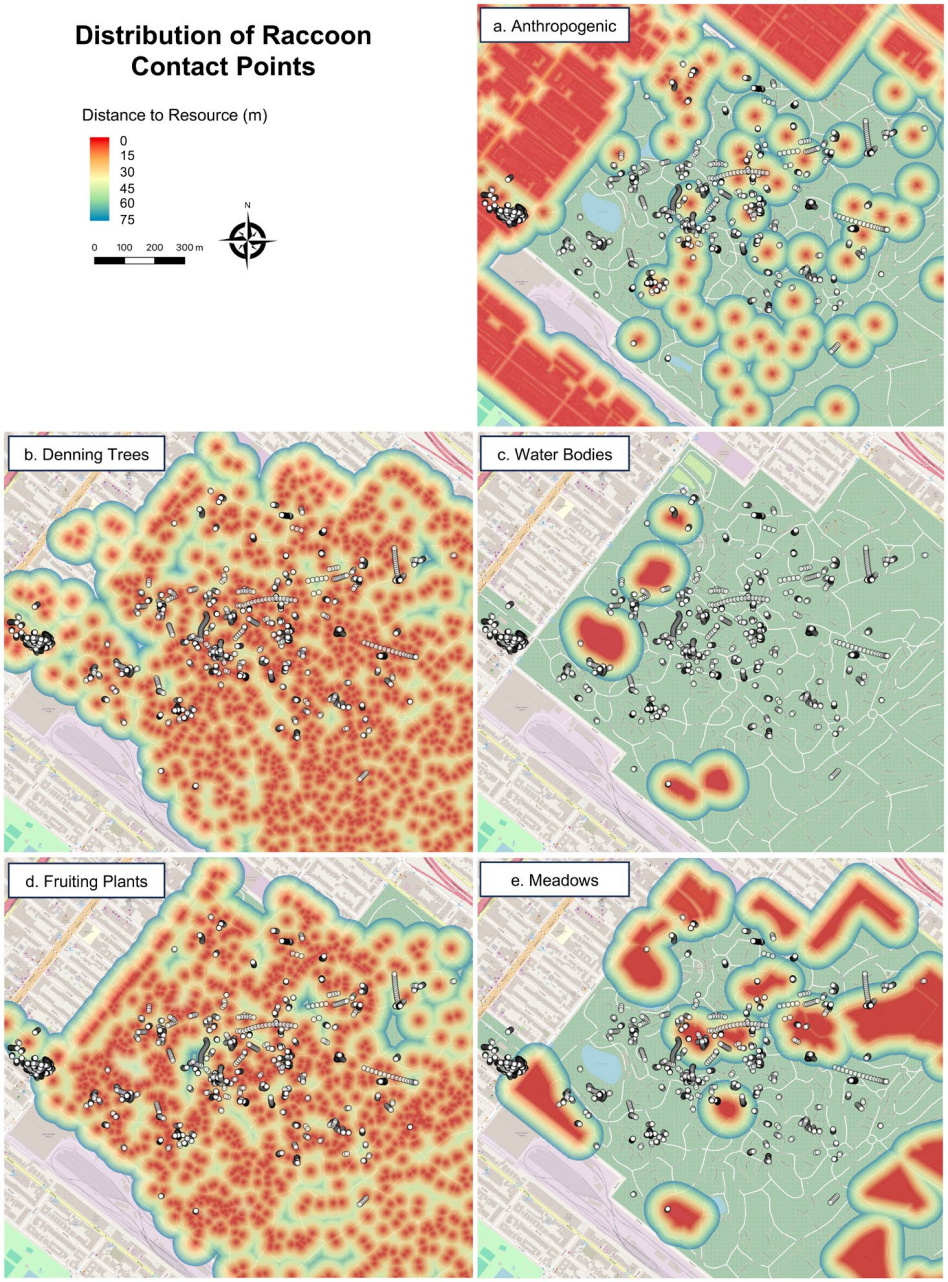
Distribution of 3251 contact points between 38 raccoon dyads across the study site. Inferred contact points (white dots with black outline) are overlaid on a map of distance to each of the nearest resource types included in the contact RSF models (a, distance to nearest anthropogenic feature, b, distance to nearest denning tree, c, distance to nearest water body, d, distance to nearest fruiting plant, e, distance to nearest meadow). All distance maps were clipped to a limit of 75 m thus distances > 75 m to each resource type are not shown.

We identified 3336 possible contact points across 52 raccoon dyads involving 18 individuals (mean = 64.15 points per dyad, minimum = 2 points per dyad, maximum = 793 points per dyad) (Table S2). The 3336 contact points occurred across 366 contact events with raccoon dyads on average experiencing 7.04 contact events (minimum = 1 event per dyad, maximum = 75 events per dyad). To ensure reliable statistical inference and address singularity issues, we only included raccoon dyads with at least 10 contact points representing at least two contact events in the RSF model. Thus, the RSF dataset included 3251 contacts across 38 raccoon dyads involving 15 individuals (mean = 85.55 points per dyad, minimum = 11 points per dyad, maximum = 793 points per dyad). The 3251 contacts occurred across 349 contact events with raccoon dyads on average experiencing 9.18 events (minimum = 2 events per dyad, maximum = 75 events per dyad). The mean distance of a contact point relative to a non‐contact point to an anthropogenic resource was 52.13 m and 64.7 m, respectively. The mean distance of a contact point relative to a non‐contact point to a fruiting plant was 30.11 m and 25.03 m, respectively. The mean distance of a contact point relative to a non‐contact point to a denning tree was 14.11 m and 17.33 m, respectively. The mean distance of a contact point relative to a non‐contact point to a meadow was 86.96 m and 99.45 m, respectively. Lastly, the mean distance of a contact point relative to a non‐contact point to a water body was 277.2 m and 248.93 m, respectively. Patterning among the distribution of contact points across the study area suggested an underlying spatial pattern associated with anthropogenic resources (Figure 3a), natural resources (Figure 3d,e), and denning trees (Figure 3b).

### 3.4 Contact RSF

**FIGURE 4:**
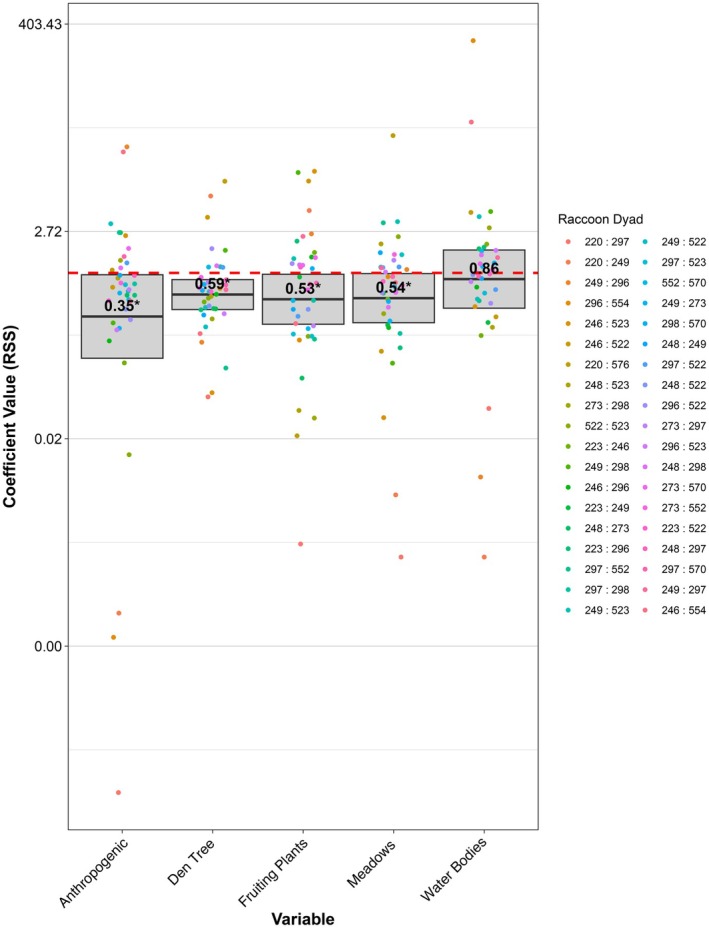
Exponentiated raccoon contact RSF coefficients. Population‐level estimates are indicated by the black line with the 95% confidence interval for each estimate represented by the gray box. Significant (*p* < 0.05) variables are indicated with an *. Dyad‐level random coefficients for each resource variable are colored by each raccoon pairing and plotted alongside population estimates.

The best fit model based on AIC included all five centered and scaled distance resource variables (distance to nearest anthropogenic resource, distance to nearest fruiting plant, distance to nearest den tree, distance to nearest water body, distance to nearest meadow; Table S3). This model included random intercepts for each raccoon dyad, a large, fixed variance for the intercept, random slopes for each of the distance variables for each dyad, and assigned a weight of 1000 to available (non‐contact) points and 1 to used (contact) points. At the population level, four (anthropogenic, fruiting plant, denning trees, meadows) of the five resources had a significant effect (*p* < 0.05) on the probability of observing a contact point given the underlying use of each resource by raccoons, unrelated to contact (Table 1). Raccoons were 2.86× more likely to co‐occur at a location one standard deviation closer to an anthropogenic resource relative to their underlying use of anthropogenic features (Figure 4; RSS Anthropogenic: 0.35 [0.013–0.96]). Raccoons were also 1.7×, 1.9×, and 1.85× more likely to co‐occur at a location one standard deviation closer to a denning tree, a fruiting plant, and a meadow, respectively (Figure 4; RSS, Denning Tree: 0.59 [0.41–0.85]; RSS, Fruit/Berry: 0.53 [0.29–0.97]; RSS Meadow: 0.54 [0.3–0.98]). Raccoons were also 1.16× more likely to co‐occur closer to water bodies; however, the 95% confidence intervals overlapped 1 (Figure 4; RSS Water Bodies: 0.86 [0.43–74]) and the effect was not significant, indicating that water bodies do not influence co‐occurrence relative to raccoon use of water bodies as a resource. Thus, the findings suggest that proximity to anthropogenic resources, denning trees, natural resources such as fruit and berry producing plants, and meadows significantly increases the likelihood of spatiotemporal co‐occurrence among raccoons, beyond what would be expected based on resource use alone. While there is a marked effect of these resources, in particular anthropogenic resources, on raccoon co‐occurrence at the population level, variability among dyad‐level coefficients (Figure 4) suggests that the effect of these resources differed between raccoon pairings.

**TABLE 1:** Result from top contact RSF model, un‐exponentiated.

Fixed effects	β Estimate	Standard error	*p*
Intercept	−0.002	162.209	1.00
Distance to anthropogenic resource	−1.051	0.513	0.041*
Distance to fruit/berry producing plant	−0.635	0.307	0.039*
Distance to denning tree	−0.521	0.184	0.005*
Distance to meadow	−0.609	0.302	0.044*
Distance to water body	−0.609	0.302	0.678

Random effects	Variance	Standard deviation	
Pair (intercept)	1,000,000	1000	
Distance to anthropogenic resource	9.256	3.042	
Distance to fruit/berry producing plant	3.363	1.834	
Distance to denning tree	1.186	1.089	
Distance to meadow	3.215	1.793	
Distance to water body	4.376	2.092	

*Note:* Significant variables are indicated with an *.

### 3.5 Contact Typologies

**FIGURE 5:**
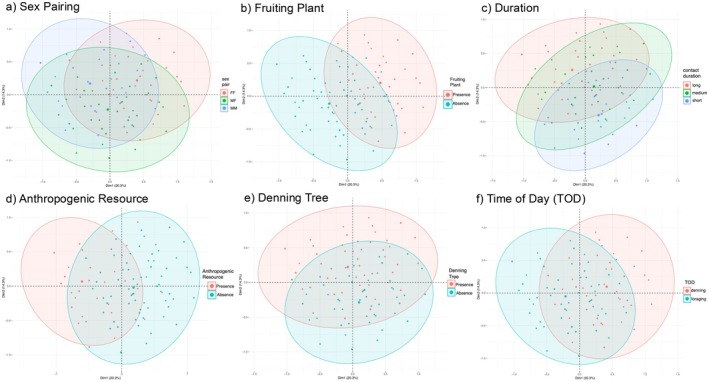
Contact events plotted on MCA axes and colored by variable category affiliation. A concentration ellipse is added around the contact events belonging to each variable category and colored by variable category.

Using the full contact point dataset (i.e., before removing data from pairs with < 10 contact points over < 2 contact events for the RSF), we identified 366 contact events, which occurred between 52 raccoon dyads representing 18 individuals (Figure S6). The first two dimensions of the MCA explained 34.6% of the variance between contact events (eigenvalue dimension 1 = 0.27, eigenvalue dimension 2 = 0.19, % variance dimension 1 = 20.29%, % variance dimension 2 = 14.31%). Anthropogenic resource presence/absence and natural (i.e., fruiting plant) resource presence/absence were most correlated with dimension 1 and denning resource presence/absence and contact event duration were most correlated with dimension 2 (Figure S4). The quality of representation (cos2), which is the squared cosine distance and indicates the confidence in the placement of the category across the first two dimensions, was greatest (cos2 > 0.4) for the variable categories anthropogenic resource presence, anthropogenic resource absence, natural resource (i.e., fruiting plant) presence, natural resource absence, long duration and short duration contact events (Figure S5a). The quality of representation for denning hours, foraging hours, presence of a denning resource, absence of a denning resource, and FF, MF, and MM contact events was slightly lower (0.25 < cos2 < 0.4) indicating that the position of these categories on the plot should be evaluated with some caution (Figure S5a). The quality of representation for medium duration contact events was the lowest (cos2 < 0.1) suggesting that a higher dimensional solution is needed to represent this category (Figure S5a). The categories anthropogenic resource presence, natural resource absence, foraging hours, and MM pairing contributed most to the negative pole of the first dimension whereas natural resource presence, FF pairings, and denning hours contributed most to the positive pole of the first dimension (Figure S5b). The categories long duration contacts, presence of a denning tree, and same‐sex pairings (MM or FF) contributed most to the positive pole of the second dimension whereas short duration contact events, MF pairings, and absence of a denning resource contributed most to the negative pole of the second dimension (Figure S5b). Clustering of variable categories on the MCA plot indicated the presence of three potential contact types across the contact event data. The first contact type (Type I) encompasses the upper right quadrant of the MCA plot (positive pole of the first dimension and positive pole of the second dimension, Figure 5). This type is associated with fruiting plants and occurs predominantly between FF pairs early or late in the night when raccoons may be emerging from or returning to their dens (denning hours) (Figure 5a,b,e,f). In addition, this contact type is negatively associated with the absence of fruiting plants and peak foraging hours (Figure 5b,f). The second contact type (Type II) is defined by the upper left quadrant of the MCA plot (negative pole of the first dimension and positive pole of the second dimension, Figure 5). These interactions are associated with the presence of anthropogenic resources, occur predominantly among MM pairs, and tend to be longer (Figure 5a,c,d). This contact type is negatively associated with short duration interactions (Figure 5a,c). The third type (Type III) is defined by the lower right quadrant of the MCA plot (positive pole of the first dimension and negative pole of the second dimension, Figure 5). These contacts are among MF pairings, shorter in duration, and not associated with any resources (Figure 5a,c).

## 4 Discussion

### 4.1 Urban Raccoons Exhibited Reduced and Constricted Home Ranges

In urban areas, anthropogenic subsidies promote raccoon site fidelity and increase rates of annual recruitment, resulting in high‐density populations (Prange et al. 2004; Bozek et al. 2007; Graser et al. 2012). Spatial confinement and clustering of anthropogenic subsidies may also reduce urban raccoon home range sizes compared to their rural counterparts (Prange et al. 2004; Bozek et al. 2007; Gautrelet et al. 2024). For example, urban raccoon home range sizes reported by Prange et al. (2004) range between 25.2 and 52.8 ha, compared to 71.2 and 182.4 ha for rural raccoons. In agreement with Prange et al. (2004), the raccoons in this study maintained similarly small home ranges (2.83‐ha to 76.08‐ha, mean of 32.87‐ha). We also noted fine‐scale variation within our population, with individuals that foraged in the urban matrix maintaining smaller home ranges compared to those that lived within the cemetery. Additionally, raccoons that lived within the cemetery generally remained within its boundaries—with the exception of the service yard, which acted as a permeable edge allowing access to the neighboring anthropogenic environment. When resource needs are met within an urban greenspace (e.g., natural resources and trash), exposure to hazards like traffic may deter raccoons from foraging in neighboring urban areas (Graser et al. 2012; Santonastaso et al. 2012). In our system, the risk of crossing the road between the cemetery and urban area (5th Avenue, a busy thoroughfare) may have discouraged raccoons from extending their range despite the abundance of anthropogenic resources available. For raccoons that lived in the urban matrix, their smaller home range size could be due to increased barriers to movement, such as roads and dense buildings, as well as their resource needs being met by the wealth of reliable anthropogenic subsidies.

### 4.2 Raccoon Space Use and Interactions Are Sensitive to Resource Patterning

Although high population densities among urban raccoons may increase spatial overlap broadly, we observed fine‐scale structuring in co‐occurrence patterns, indicating that individuals disproportionately shared space in specific locations within overlapping ranges. The population‐level contact RSF broadly supported predictions of the Resource Dispersion Hypothesis (RDH). Contact points were not associated with all locations but, instead, were disproportionately likely near specific resources, with relative effects aligned with each resource's predicted degree of clustering, abundance, and reliability. Proximity to anthropogenic resources had the strongest positive effect on co‐occurrence relative to natural food resources (e.g., meadows or fruiting plants) or den trees. Although anthropogenic resources are often located within or adjacent to the high‐risk urban matrix, their energy content, abundance, and predictability can make them attractive to raccoons. Prior studies have documented frequent raccoon use of dumpsters, compost, and intentional cat feeding stations in urban areas, particularly on the periphery of green patches (Totton et al. 2002; Wright and Gompper 2005; Bozek et al. 2007; Crandall et al. 2024). Consistent with RDH, anthropogenic resources at our site may have reduced the costs of spatial overlap and promoted tolerance, facilitating temporary aggregations (Carr and Macdonald 1986; Johnson et al. 2002; Macdonald and Johnson 2015). For example, Wehtje and Gompper (2011) found that clumped food sources increased home range overlap, particularly among females, and Prange et al. (2011) identified fission‐fusion dynamics among urban raccoons, with male sociality linked to variation in resource abundance and its effects on female distribution. Our findings extend this work by showing that anthropogenic resources not only attract raccoons but also facilitate simultaneous use, serving as potential contact hotspots beyond what would be expected from space use alone and supporting a mechanism of tolerance‐based aggregation consistent with the RDH framework.

Contact points were also significantly associated with proximity to natural resources, including den trees, fruiting plants, and meadows. These features likely supported transient or context‐dependent associations. During rearing, adult female raccoons share dens with their young (Mech and Turkowski 1966; Endres and Smith 1993; Hauver, Gehrt, and Prange 2010; Smith and Endres 2012). Winter co‐denning also often occurs between unrelated individuals for thermoregulation and is associated with increased rates of contact and pathogen transmission (Robert et al. 2013; Hirsch et al. 2016). Our study took place during the summer/fall when co‐denning is less common, and we only tracked adults; thus, the reduced effect of den trees in our models may reflect seasonal reductions in communal denning and missed interactions at maternal dens. At our site, fruiting plants may have facilitated transient foraging overlap, especially during the summer/fall when soft mast availability is high. Meadows may also support invertebrate foraging, potentially promoting opportunistic co‐use. However, the weaker effect of these features relative to anthropogenic resources may reflect their more diffuse distribution, temporal variability, or stronger competition costs (Beasley and Rhodes 2010; Beasley et al. 2011; Monello and Gompper 2011; Wehtje and Gompper 2011; Graser et al. 2012). Notably, despite reported relevance to raccoon resource selection (Urban 1970; Henner et al. 2004; Bozek et al. 2007; Beasley and Rhodes 2010; Heske and Ahlers 2016), water bodies had no effect on co‐occurrence, possibly due to their broader spatial distribution at our site or asynchronous use.

### 4.3 Characteristics of Urban Raccoon Contact Events Reflect Underlying Socio‐Spatial Factors

Raccoon pairwise contact RSF coefficient estimates showed high variability in dyad‐level responses to different resources. Results from the MCA indicated that this inter‐pair variability may be explained by different types of co‐occurrence (i.e., contact typologies) among raccoons defined by sex, time of day, and presence of different resources. We detected structure in the contact event data characterized by: (1) longer co‐occurrences among males near anthropogenic resources, (2) female co‐occurrence around natural resources and while denning (dusk/dawn), and (3) transient proximity not associated with any resource among male–female dyads. The characteristics of each type may reflect underlying socio‐spatial factors (e.g., resource‐driven aggregation, sex‐based differences in space use) that jointly influence where, when, and how individuals interact.

In our MCA, male–male dyads were associated with longer‐duration contact events near anthropogenic resources. Although we observed fewer male–male pairings (MM: 6 dyads, MF: 28 dyads, FF: 18 dyads), they accounted for a substantial number of contact events overall (MM: 106 contacts, MF: 169 contacts, FF: 91 contacts), suggesting repeated co‐occurrences among a subset of males. This pattern aligns with RDH‐driven aggregation around high‐value resources and may also reflect sex‐specific social structure and behavioral flexibility in urban raccoons. Urban raccoons exhibit sex‐structured social networks and fission‐fusion dynamics, with males more likely to form repeated associations and females remaining solitary, particularly outside of mating (Gehrt et al. 2008; Hauver, Gehrt, Prange, and Dubach 2010; Prange et al. 2011; Hirsch et al. 2013a, 2013b; Robert et al. 2013; Reynolds et al. 2015). For example, Reynolds et al. (2015) found that male–male raccoon interactions during the non‐breeding season were most frequent, longest in duration, and often involved consistent pairings, indicating stable associations. These dynamics may reflect the effects of clustered urban resources that support high densities and overlapping female ranges, reducing the utility of territoriality, favoring promiscuity, and making tolerance‐based male groups advantageous for accessing both mates and high‐value patches (Gehrt and Fritzell 1998b; Chamberlain and Leopold 2002; Prange et al. 2003, 2011; Bozek et al. 2007; Hauver, Gehrt, Prange, and Dubach 2010; Hauver et al. 2013; Hirsch et al. 2013b; Schuttler et al. 2015). While not evidence of social bonds, our observation of repeated, prolonged male–male co‐occurrence near anthropogenic resources is consistent with tolerance‐based associations shaped by anthropogenic resource distribution and potentially, male social behavior.

Female–female contact events were less common and, when they occurred, were near natural resources (e.g., fruiting plants, den trees) and during dusk/dawn when denning activity is likely. Female raccoon distribution, particularly during the rearing season, is shaped by den (e.g., tree cavities) and resource availability (Beasley and Rhodes 2010, 2012; Beasley et al. 2011). While females are typically solitary in natural settings (Fritzell 1978; Gehrt and Fritzell 1998b; Chamberlain and Leopold 2002; Pitt et al. 2008a), clustered resources and high‐density populations in cities may facilitate more frequent, transient interactions related to amicable sharing of space, especially during rearing when den and resource needs increase (Prange et al. 2011; Robert et al. 2013). The location and timing of female–female contact events at our site suggest associations were more situational and likely reflect tolerance among neighboring females related to den and resource needs and perhaps avoidance of male‐dominated areas while with young. Male–female contact events were shortest in duration and not associated with any resource type. While a male group's range typically overlaps with several females to ensure mate access, interactions are often constrained to the mating season (winter) (Henner et al. 2004; Prange et al. 2004, 2011; Hirsch et al. 2013a, 2013b; Reynolds et al. 2015). Our study occurred during the rearing season, when females with young may avoid males (Hauver, Gehrt, and Prange 2010); thus, our observed pattern likely reflects these seasonal dynamics with brief, incidental male–female co‐occurrences arising from spatial overlap rather than structured association or even tolerance.

### 4.4 Implications for Pathogen Transmission

Our research suggests that anthropogenic resources facilitate contact opportunities among urban raccoons and may interact with raccoon social dynamics (e.g., male groups) to shape the frequency and distribution of contacts across the landscape with implications for pathogen transmission in cities. When resources are unevenly distributed (such as in cities), non‐group living animals may aggregate in resource‐rich areas and show greater tolerance for conspecifics, which alters rates of contact and impacts associated processes like pathogen transmission (Hirsch et al. 2013b; Becker and Hall 2014; Becker et al. 2015, 2018). For example, among raccoons, concentrated food resources have been linked to higher infection rates with endoparasites due to increased rates of co‐occurrence (Wright and Gompper 2005). Simulation studies also indicate that outbreak dynamics in raccoons are influenced by seasonal social behaviors—rabies outbreaks tend to be larger when introduced mid‐way through the non‐breeding season and spread fastest when introduced at the start of the breeding season (Reynolds et al. 2015). While our study was limited to the non‐breeding season when contact rates are lower compared to the breeding season, we still observed frequent, prolonged contact opportunities among males near anthropogenic resources, brief male–female co‐occurrences, and few female–female interactions tied to denning.

These findings suggest that if a pathogen were introduced at this time, transmission chains may be male‐dominated. In the absence of inter‐male group connectivity and prolonged male–female interactions, a pathogen may fail to spread despite a high‐density population. However, in the fall, juveniles become more independent and may begin dispersing, which could increase connectivity among male groups and solitary females and facilitate persistence to the winter, when contact rates increase (Gehrt and Fritzell 1998a; Cullingham et al. 2008; Hisey 2014). Our models also highlighted that foraging‐related features may facilitate raccoon spatiotemporal overlap. Thus, increased foraging during the fall may also elevate the frequency of incidental, transient interactions, as we observed among male–female pairs (Pitt et al. 2008b). While not conducive to the spread of pathogens like rabies, which require close contact (e.g., biting), these interactions could contribute to the spread of highly infectious, aerosolized pathogens with long infection windows (e.g., canine distemper virus, CDV) that are becoming an increasing problem among urban and peri‐urban wildlife.

### 4.5 Limitations

Together, our results indicate that urban raccoon co‐occurrence is not just a passive consequence of overlapping space use in a high‐density population, but instead reflects spatially heterogeneous patterns shaped by selective tolerance likely emerging from socio‐spatial responses to urban life. However, some caution should be taken in interpreting these results. Co‐occurrences were inferred from interpolated GPS trajectories and were not directly observed. Although raccoons perceive conspecifics through olfaction and vision at distances likely exceeding our 15 m threshold (Kent and Tang‐Martínez 2014; Morton 2021; Buzuleciu et al. 2016), we did not confirm the nature or outcome of each event. Instead, we interpret co‐occurrence as a proxy for contact opportunity, which, while necessary for interaction, is not definitive of it. Thus, we caution interpreting these findings as evidence of social structure or affiliative behavior. Instead, our results are consistent with patterns that would be expected if such dynamics, as reported by others, were present. Nevertheless, because the primary concern at our site was transmission of a highly infectious, aerosolized pathogen (CDV), brief spatial proximity or short‐lagged use of shared resources may still meaningfully contribute to transmission risk, even in the absence of direct or affiliative interaction.

## Author Contributions

**Laura Dudley Plimpton:** conceptualization (lead), formal analysis (lead), investigation (lead), methodology (lead), writing – original draft (lead). **Meredith VanAcker:** formal analysis (equal), methodology (equal), writing – review and editing (equal). **Sara Kross:** conceptualization (equal), investigation (equal), methodology (equal), writing – review and editing (equal). **Ximena A. Olarte‐Castillo:** conceptualization (equal), methodology (equal), writing – review and editing (equal). **Sara Evans:** investigation (equal), methodology (equal), writing – review and editing (equal). **Christopher R. Konowal:** investigation (equal), writing – review and editing (equal). **Meggan Craft:** conceptualization (equal), methodology (equal), writing – review and editing (equal). **Laura B. Goodman:** conceptualization (equal), methodology (equal), writing – review and editing (equal). **Gary Whittaker:** conceptualization (equal), methodology (equal), writing – review and editing (equal). **David Needle:** methodology (equal), writing – review and editing (equal). **Maria Diuk‐Wasser:** conceptualization (lead), investigation (lead), methodology (lead), resources (lead), supervision (lead), writing – original draft (equal), writing – review and editing (equal).

## Funding

The authors have nothing to report.

## Disclosure

Statement of Inclusion: Our study was conducted locally with the invaluable support and input of neighboring residents and staff, workers, and visitors of The Green‐Wood Cemetery. This work incorporated interdisciplinary collaboration across wildlife ecology, molecular biology, technology, horticulture, public health, and veterinary sciences. We worked closely with local stakeholders including Green‐Wood Cemetery staff, neighboring residents, and the New York City Department of Health to obtain feedback on research aims, sampling procedures, and communication approaches. We continue to share our findings through regular research update meetings with representatives from academia, public health, and Green‐Wood. We are also committed to continuing to explore opportunities to engage with the surrounding community and to communicate results in accessible formats that highlight the implications of this work for urban wildlife management.

## Conflicts of Interest

The authors declare no conflicts of interest.

## Supporting information


**Appendix S1:** ece372559‐sup‐0001‐AppendixS1.docx.

**Appendix S2:** ece372559‐sup‐0002‐AppendixS2.docx.

## Data Availability

Data including all contact and non‐contact points and associated extracted covariates used for the contact RSF analyses and the csv of contact events and associated variables used for the contact typology analysis (MCA) are available on Dryad Digital Repository. An R code file for running each analysis is also provided. Dataset DOI: 10.5061/dryad.rxwdbrvm3.
