# Supplementary material for: Trash Talking: Anthropogenic Resources Facilitate Raccoon Interactions in Urban Environments
Source: Ecol Evol. 2025 Dec 8;15(12):e72559. doi: 10.1002/ece3.72559 (PMC12685763; doi:10.1002/ece3.72559)
Supplement: Supplementary file 1 — Appendix S1: ece372559‐sup‐0001‐AppendixS1.docx. [file ECE3-15-e72559-s001.docx]

| **Raccoon ID** | **Sex** | **HR size, (95% wAKDEc, ha)** | **Monitoring Duration (days)** | **# Relocations** | **Crossing Time (hrs)** | **Movement Model** |
| --- | --- | --- | --- | --- | --- | --- |
| RA-220 | F | 37.85 (28.7, 48.24) | 28.26 | 378 | 2.06 | OUF Anisotropic |
| RA-223 | M | 31.45 (20.98-44.01) | 20.22 | 266 | 8.55 | OU anisotropic |
| RA-244 | M | 15.63 (10.8, 21.34) | 4.96 | 168 | 1.46 | OUF |
| RA-246 | M | 2.83 (2.48, 3.2) | 38.4 | 471 | 1.06 | OU anisotropic |
| RA-248 | M | 32.3 (26.65, 38.44) | 29.3 | 925 | 4.11 | OUF |
| RA-249 | F | 55.14 (48.61, 62.1) | 34.5 | 1022 | 1.66 | OUF anisotropic |
| RA-273 | M | 40.42 (37.3, 43.68) | 88.97 | 2055 | 1.38 | OUF anisotropic |
| RA-296 | F | 34.06 (26.53, 42.52) | 20.96 | 480 | 3.85 | OUF anisotropic |
| RA-297 | F | 54.87 (46.1, 64.38) | 61.09 | 1045 | 6.00 | OUF anisotropic |
| RA-298 | F | 17.48 (14.66, 20.54) | 33.02 | 387 | 2.09 | OUF |
| RA-522 | F | 29.42 (25.4, 33.75) | 27.39 | 807 | 1.67 | OUF anisotropic |
| RA-523 | F | 34.4 (26.87, 42.95) | 21.21 | 227 | 2.64 | OUF anisotropic |
| RA-551 | M | 19.7 (16.95, 22.65) | 54.04 | 839 | 2.61 | OUF anisotropic |
| RA-552 | F | 50.8 (43.3, 58.9) | 23.96 | 842 | 1.88 | OUF anisotropic |
| RA-554 | M | 5.18 (4.44, 5.99) | 48.5 | 418 | 1.61 | OU |
| RA-570 | M | 76.08 (68.7, 83.81) | 78.42 | 1802 | 2.59 | OUF anisotropic |
| RA-576 | F | 13.17 (9.16, 17.89) | 9.25 | 238 | 3.05 | OU anisotropic |
| RA-580 | F | 25.92 (18.5, 34.6) | 12.33 | 233 | 2.89 | OUF |
| RA-6843 | M | 42.2 (34.15, 51.54) | 22.89 | 367 | 2.20 | OU anisotropic |

**Supplement, Table 1.** *wAKDEc Analysis for 19 NYC Raccoons.* Sex, home range size (95% wAKDEc with 95% confidence intervals), number of monitoring days, number of relocations, home range crossing time, and movement model based on *ctmm* analysis.

**Supplement, Table 2.** *Raccoon Contact Data.* Sex pairing, overlapping collaring time, number of contact points, and number of contact events observed across all 171 possible unique pairings/dyads of the 19 monitored raccoons. In total 53 pairs experienced at least 1 contact. Raccoon pairings that were not collared at the same time are indicated by an overlapping collaring time of 0 and NA for # of contact pts and # of contact events. Pairs are ordered from the greatest number of contact points observed to least.

| **Raccoon Dyad** | **Sex Pairing** | **Overlapping Collaring Time (hrs)** | **# Contact Pts** | **# Contact Events** |
| --- | --- | --- | --- | --- |
| 273 : 570 | MM | 1882.066667 | 793 | 75 |
| 223 : 296 | MF | 367.4833333 | 359 | 25 |
| 223 : 246 | MM | 485.2833333 | 228 | 20 |
| 273 : 297 | MF | 1465.75 | 177 | 23 |
| 296 : 522 | FF | 502.9166667 | 155 | 11 |
| 246 : 296 | MF | 502.9166667 | 145 | 14 |
| 249 : 297 | FF | 774.5833333 | 133 | 11 |
| 522 : 523 | FF | 509.1333333 | 109 | 11 |
| 248 : 297 | MF | 655.15 | 93 | 16 |
| 223 : 522 | MF | 415.5 | 92 | 9 |
| 220 : 576 | FF | 221.9166667 | 88 | 4 |
| 297 : 522 | FF | 654.8333333 | 86 | 7 |
| 249 : 522 | FF | 534.5333333 | 63 | 7 |
| 248 : 249 | MF | 702.85 | 60 | 10 |
| 246 : 554 | MM | 918.3833333 | 54 | 5 |
| 248 : 298 | MF | 655.1333333 | 54 | 7 |
| 249 : 273 | MF | 774.2666667 | 52 | 10 |
| 249 : 296 | FF | 486.5166667 | 51 | 7 |
| 248 : 273 | MM | 654.8333333 | 42 | 4 |
| 297 : 298 | FF | 792.35 | 38 | 5 |
| 246 : 522 | MF | 634.55 | 36 | 2 |
| 246 : 523 | MF | 509.1333333 | 33 | 4 |
| 223 : 249 | MF | 485.2833333 | 30 | 6 |
| 297 : 570 | MF | 1226.05 | 29 | 7 |
| 273 : 298 | MF | 792.05 | 27 | 3 |
| 297 : 523 | FF | 509.1333333 | 27 | 6 |
| 273 : 552 | MF | 574.95 | 20 | 7 |
| 248 : 523 | MF | 389.7166667 | 20 | 2 |
| 249 : 298 | FF | 774.5666667 | 18 | 2 |
| 220 : 249 | FF | 606.3666667 | 18 | 2 |
| 552 : 570 | MF | 574.95 | 18 | 3 |
| 298 : 570 | MF | 552.35 | 17 | 3 |
| 296 : 554 | MF | 502.9166667 | 17 | 3 |
| 220 : 297 | FF | 678.6 | 16 | 2 |
| 297 : 552 | FF | 574.95 | 14 | 5 |
| 249 : 523 | FF | 509.1333333 | 14 | 5 |
| 296 : 523 | FF | 486.5 | 14 | 3 |
| 273 : 523 | MF | 509.1333333 | 13 | 1 |
| 248 : 522 | MF | 415.1 | 11 | 3 |
| 552 : 580 | FF | 47.7 | 11 | 1 |
| 273 : 522 | MF | 654.8333333 | 9 | 1 |
| 551 : 580 | MF | 295.7833333 | 9 | 1 |
| 523 : 570 | MF | 509.1333333 | 7 | 2 |
| 552 : 843 | MF | 336.7666667 | 6 | 2 |
| 580 : 843 | MF | 247.6666667 | 6 | 1 |
| 220 : 552 | FF | 246.0333333 | 6 | 1 |
| 223 : 523 | MF | 390.1166667 | 5 | 2 |
| 223 : 297 | MF | 485.2833333 | 3 | 1 |
| 223 : 554 | MM | 485.2833333 | 3 | 1 |
| 248 : 570 | MM | 415.1333333 | 3 | 1 |
| 248 : 296 | MF | 367.0833333 | 2 | 1 |
| 552 : 576 | FF | 221.1333333 | 2 | 1 |
| 273 : 551 | MM | 1296.883333 | 0 | 0 |
| 551 : 570 | MM | 1296.883333 | 0 | 0 |
| 297 : 554 | MF | 1114.816667 | 0 | 0 |
| 273 : 554 | MM | 1114.5 | 0 | 0 |
| 554 : 570 | MM | 874.8 | 0 | 0 |
| 246 : 297 | MF | 874.6 | 0 | 0 |
| 246 : 273 | MM | 874.2833333 | 0 | 0 |
| 297 : 551 | MF | 858.9666667 | 0 | 0 |
| 246 : 249 | MF | 821.6666667 | 0 | 0 |
| 249 : 554 | MF | 818.3666667 | 0 | 0 |
| 246 : 298 | MF | 792.35 | 0 | 0 |
| 298 : 554 | MF | 792.35 | 0 | 0 |
| 246 : 248 | MM | 702.2333333 | 0 | 0 |
| 248 : 554 | MM | 698.9333333 | 0 | 0 |
| 220 : 246 | MF | 678.6 | 0 | 0 |
| 220 : 273 | MF | 678.6 | 0 | 0 |
| 220 : 554 | MF | 678.6 | 0 | 0 |
| 522 : 554 | MF | 654.8333333 | 0 | 0 |
| 522 : 570 | MF | 654.8333333 | 0 | 0 |
| 246 : 570 | MM | 634.5833333 | 0 | 0 |
| 220 : 298 | FF | 624.15 | 0 | 0 |
| 220 : 570 | MF | 606.8 | 0 | 0 |
| 220 : 522 | FF | 606.7666667 | 0 | 0 |
| 551 : 552 | MF | 568.6333333 | 0 | 0 |
| 298 : 522 | FF | 552.3166667 | 0 | 0 |
| 273 : 843 | MM | 536.7333333 | 0 | 0 |
| 297 : 843 | MF | 536.7333333 | 0 | 0 |
| 551 : 843 | MM | 536.7333333 | 0 | 0 |
| 570 : 843 | MM | 536.7333333 | 0 | 0 |
| 249 : 570 | MF | 534.5666667 | 0 | 0 |
| 552 : 554 | MF | 514.0333333 | 0 | 0 |
| 220 : 523 | FF | 509.1333333 | 0 | 0 |
| 298 : 523 | FF | 509.1333333 | 0 | 0 |
| 523 : 554 | MF | 509.1333333 | 0 | 0 |
| 551 : 554 | MM | 507.7166667 | 0 | 0 |
| 220 : 296 | FF | 502.9166667 | 0 | 0 |
| 273 : 296 | MF | 502.9166667 | 0 | 0 |
| 296 : 297 | FF | 502.9166667 | 0 | 0 |
| 296 : 298 | FF | 502.9166667 | 0 | 0 |
| 296 : 570 | MF | 502.9166667 | 0 | 0 |
| 220 : 248 | MF | 486.9333333 | 0 | 0 |
| 220 : 223 | MF | 485.2833333 | 0 | 0 |
| 223 : 273 | MM | 485.2833333 | 0 | 0 |
| 223 : 298 | MF | 485.2833333 | 0 | 0 |
| 223 : 248 | MM | 484.8833333 | 0 | 0 |
| 223 : 570 | MM | 415.5333333 | 0 | 0 |
| 273 : 580 | MF | 295.7833333 | 0 | 0 |
| 297 : 580 | FF | 295.7833333 | 0 | 0 |
| 570 : 580 | MF | 295.7833333 | 0 | 0 |
| 522 : 552 | FF | 294.1 | 0 | 0 |
| 522 : 551 | MF | 287.7833333 | 0 | 0 |
| 554 : 843 | MM | 275.85 | 0 | 0 |
| 246 : 552 | MF | 273.8166667 | 0 | 0 |
| 246 : 551 | MM | 267.5 | 0 | 0 |
| 220 : 551 | MF | 239.7166667 | 0 | 0 |
| 246 : 576 | MF | 221.9166667 | 0 | 0 |
| 273 : 576 | MF | 221.9166667 | 0 | 0 |
| 297 : 576 | FF | 221.9166667 | 0 | 0 |
| 522 : 576 | FF | 221.9166667 | 0 | 0 |
| 554 : 576 | MF | 221.9166667 | 0 | 0 |
| 570 : 576 | MF | 221.9166667 | 0 | 0 |
| 551 : 576 | MF | 214.8166667 | 0 | 0 |
| 298 : 576 | FF | 192.3666667 | 0 | 0 |
| 298 : 552 | FF | 191.5833333 | 0 | 0 |
| 296 : 576 | FF | 190.9833333 | 0 | 0 |
| 296 : 552 | FF | 190.2 | 0 | 0 |
| 298 : 551 | MF | 185.2666667 | 0 | 0 |
| 296 : 551 | MF | 183.8833333 | 0 | 0 |
| 249 : 576 | FF | 174.5833333 | 0 | 0 |
| 523 : 576 | FF | 174.5666667 | 0 | 0 |
| 249 : 552 | FF | 173.8 | 0 | 0 |
| 523 : 552 | FF | 173.7833333 | 0 | 0 |
| 249 : 551 | MF | 167.4833333 | 0 | 0 |
| 523 : 551 | MF | 167.4666667 | 0 | 0 |
| 244 : 273 | MM | 119 | 0 | 0 |
| 244 : 297 | MF | 119 | 0 | 0 |
| 244 : 551 | MM | 119 | 0 | 0 |
| 244 : 552 | MF | 119 | 0 | 0 |
| 244 : 570 | MM | 119 | 0 | 0 |
| 244 : 843 | MM | 119 | 0 | 0 |
| 244 : 554 | MM | 106.85 | 0 | 0 |
| 522 : 843 | MF | 55.91666667 | 0 | 0 |
| 223 : 576 | MF | 55.55 | 0 | 0 |
| 248 : 576 | MF | 55.15 | 0 | 0 |
| 223 : 552 | MF | 54.76666667 | 0 | 0 |
| 248 : 552 | MF | 54.36666667 | 0 | 0 |
| 223 : 551 | MM | 48.45 | 0 | 0 |
| 248 : 551 | MM | 48.05 | 0 | 0 |
| 246 : 843 | MM | 35.63333333 | 0 | 0 |
| 220 : 843 | MF | 7.85 | 0 | 0 |
| 220 : 244 | MF | 0 | NA | NA |
| 220 : 580 | FF | 0 | NA | NA |
| 223 : 244 | MM | 0 | NA | NA |
| 223 : 580 | MF | 0 | NA | NA |
| 223 : 843 | MM | 0 | NA | NA |
| 244 : 246 | MM | 0 | NA | NA |
| 244 : 248 | MM | 0 | NA | NA |
| 244 : 249 | MF | 0 | NA | NA |
| 244 : 296 | MF | 0 | NA | NA |
| 244 : 298 | MF | 0 | NA | NA |
| 244 : 522 | MF | 0 | NA | NA |
| 244 : 523 | MF | 0 | NA | NA |
| 244 : 576 | MF | 0 | NA | NA |
| 244 : 580 | MF | 0 | NA | NA |
| 246 : 580 | MF | 0 | NA | NA |
| 248 : 580 | MF | 0 | NA | NA |
| 248 : 843 | MM | 0 | NA | NA |
| 249 : 580 | FF | 0 | NA | NA |
| 249 : 843 | MF | 0 | NA | NA |
| 296 : 580 | FF | 0 | NA | NA |
| 296 : 843 | MF | 0 | NA | NA |
| 298 : 580 | FF | 0 | NA | NA |
| 298 : 843 | MF | 0 | NA | NA |
| 522 : 580 | FF | 0 | NA | NA |
| 523 : 580 | FF | 0 | NA | NA |
| 523 : 843 | MF | 0 | NA | NA |
| 554 : 580 | MF | 0 | NA | NA |
| 576 : 580 | FF | 0 | NA | NA |
| 576 : 843 | MF | 0 | NA | NA |

**Supplement, Table 3.** *Top Contact RSF Models.* 133 model structures with different combinations of the 5 raccoon resources were run. The top model and next 5 most parsimonious models based on AIC are listed below. All models included a large, fixed intercept variance and weights of 1000 applied to available (non-contact) locations and 1 for used (contact) locations. Each resource was included in the model as distance to the closest feature of that resource type (e.g., anthro.dist indicated the distance to the nearest anthropogenic resource feature) and model structures tested the improvement made by adding/dropping different resource variables, scaling and centering the variables (indicated by .s) and additionally, log transforming and then centering and scaling the distance variables (indicated by .ls).

| **Model #** | **Structure** | **AIC** | **ΔAIC** |
| --- | --- | --- | --- |
| 119 | case_ ~ anthro.dist.s + fruit.dist.s + den.dist.s + meadow.dist.s + water.dist.s + (1\|pair) + (0+anth.dist.s\|pair) (0+fruit.dist.s\|pair) + (0+den.dist.s\|pair) (0+meadow.dist.s\|pair) + (0+water.dist.s\|pair) | 63062.83 | 0 |
| 128 | case_ ~ anth.dist.s + fruit.dist.s + den.dist.s + meadow.dist.s + water.dist.ls + (1\|pair) + (0+anth.dist.s\|pair) + (0+fruit.dist.s\|pair) + (0+den.dist.s\|pair) + (0+meadow.dist.s\|pair) + (0+water.dist.ls\|pair | 63136.40 | 73.57 |
| 98 | case_ ~ anth.dist.s + fruit.dist.s + den.dist.s + meadow.dist.s + (1\|pair) + (0+anth.dist.s\|pair) + (0+fruit.dist.s\|pair) + (0+den.dist.s\|pair) + (0+meadow.dist.s\|pair | 63276.92 | 214.09 |
| 126 | case_ ~ anth.dist.s + fruit.dist.ls + den.dist.s + meadow.dist.s + water.dist.s + (1\|pair) + (0+anth.dist.s\|pair) + (0+fruit.dist.ls\|pair) + (0+den.dist.s\|pair) + (0+meadow.dist.s\|pair) + (0+water.dist.s\|pair)" | 63331.12 | 268.29 |
| 125 | case_ ~ anth.dist.ls + fruit.dist.s + den.dist.s + meadow.dist.s + water.dist.s + (1\|pair) + (0+anth.dist.ls\|pair) + (0+fruit.dist.s\|pair) + (0+den.dist.s\|pair) + (0+meadow.dist.s\|pair) + (0+water.dist.s\|pair | 63363.83 | 301 |
| 129 | case_ ~ anth.dist.ls + fruit.dist.ls + den.dist.s + meadow.dist.s + water.dist.s + (1\|pair) + (0+anth.dist.ls\|pair) + (0+fruit.dist.ls\|pair) + (0+den.dist.s\|pair) + (0+meadow.dist.s\|pair) + (0+water.dist.s\|pair) | 63519.33 | 456.5 |

**Supplement, Table 4.** *Model Validation Result.* Results from 8-fold cross validation, the testing data for each fold contained the contact and non-contact from a unique set of raccoon pairing IDs (4-5 pairs) with the training dataset containing the data from the remaining raccoon pairings. A linear regression was then fit to each iteration’s calibration data to assess how well the predicted counts match the observed. The linear regression results for each iteration are shown below. R code was adapted from Bühler et al., 2023.

| **Iteration** | **Intercept** | **Slope** | **R2** | **Spearman** |
| --- | --- | --- | --- | --- |
| M1 | 0.18 | 0.08 | 0.0 | 0.5 |
| M2 | 0.34 | -0.71 | 0.16 | -0.1 |
| M3 | 0.3 | -0.52 | 0.23 | -0.6 |
| M4 | -0.08 | 1.42 | 0.6 | 1.0 |
| M5 | 0.15 | 0.25 | 0.03 | -0.1 |
| M6 | -0.02 | 1.12 | 0.28 | 0.9 |
| M7 | -0.39 | 2.94 | 0.33 | 0.7 |
| M8 | -0.33 | 2.64 | 0.45 | 0.2 |
| **Mean** | 0.019 | 0.9 | 0.27 | 0.31 |
