# Supplementary material for: Trash Talking: Anthropogenic Resources Facilitate Raccoon Interactions in Urban Environments
Source: Ecol Evol. 2025 Dec 8;15(12):e72559. doi: 10.1002/ece3.72559 (PMC12685763; doi:10.1002/ece3.72559)
Supplement: Supplementary file 2 — Appendix S2: ece372559‐sup‐0002‐AppendixS2.docx. [file ECE3-15-e72559-s002.docx]

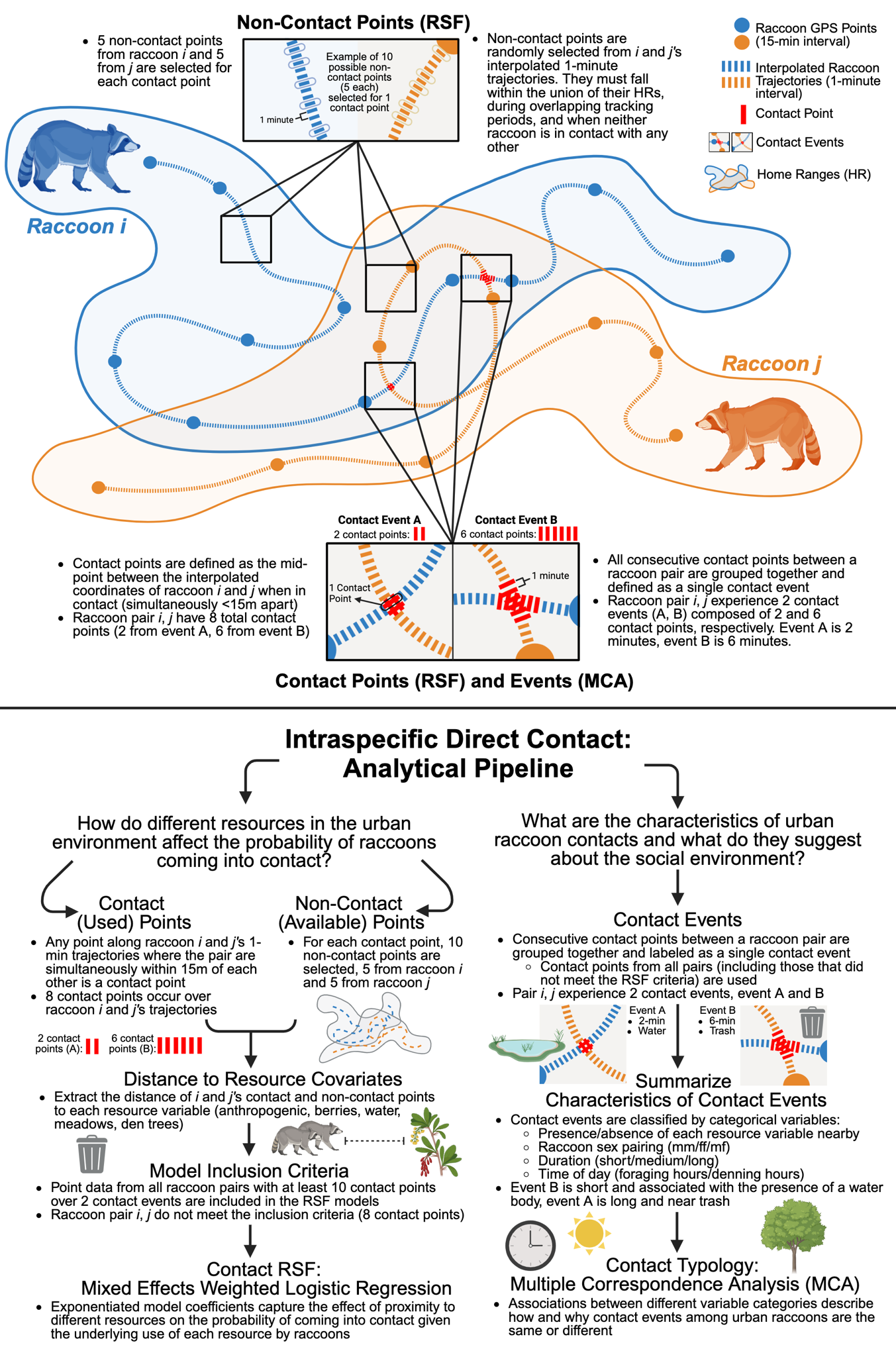


**Supplement, Figure 1.** Full schematic of raccoon contact analytical pipeline.

**Supplement, Figure 2.** *Initial location of all raccoon captures during the 2022 field season*. 32 raccoons (17M, 15F) were captured across the ~60-70-ha collaring zone over 14 trap nights (15-20 traps set per nights) between August and mid-September 2022. 19 (9M, 10F) of the 32 received collars. The initial capture locations of all 32 raccoons are shown with males in blue and females in red, collared individuals are marked with an *.

**Supplement, Figure 3.** *Distribution of* *95% wAKDEc estimates for 19 NYC raccoons.* Estimate of the 95% wAKDEc size (hectares) for each of the raccoons is plotted along with the 95% confidence intervals for each estimate.

**Supplement, Figure 4.** *Correlation between contact variables and MCA principal dimensions*. Squared correlations between variables and dimensions are used as coordinates for each variable. Presence of anthropogenic resources, presence of natural resources, and sex pairing are most correlated with dimension 1 while presence of a denning resource, sex pairing, and contact duration are most correlated with dimension 2.

**Supplement, Figure 5.** *Multiple Correspondence Analysis of 366 Raccoon Contact Events*. Dimension 1 and 2 were sufficient to retain 34.6% of the total variation within the data. (Top) Variable categories are colored by the squared cosine (cos2), which indicates the degree of association between a variable category and a particular axis. Well represented categories across the two dimensions are close to 1 and shaded in orange/red. (Bottom) Variable categories are colored by their contribution (in %) to the definition of the dimensions.


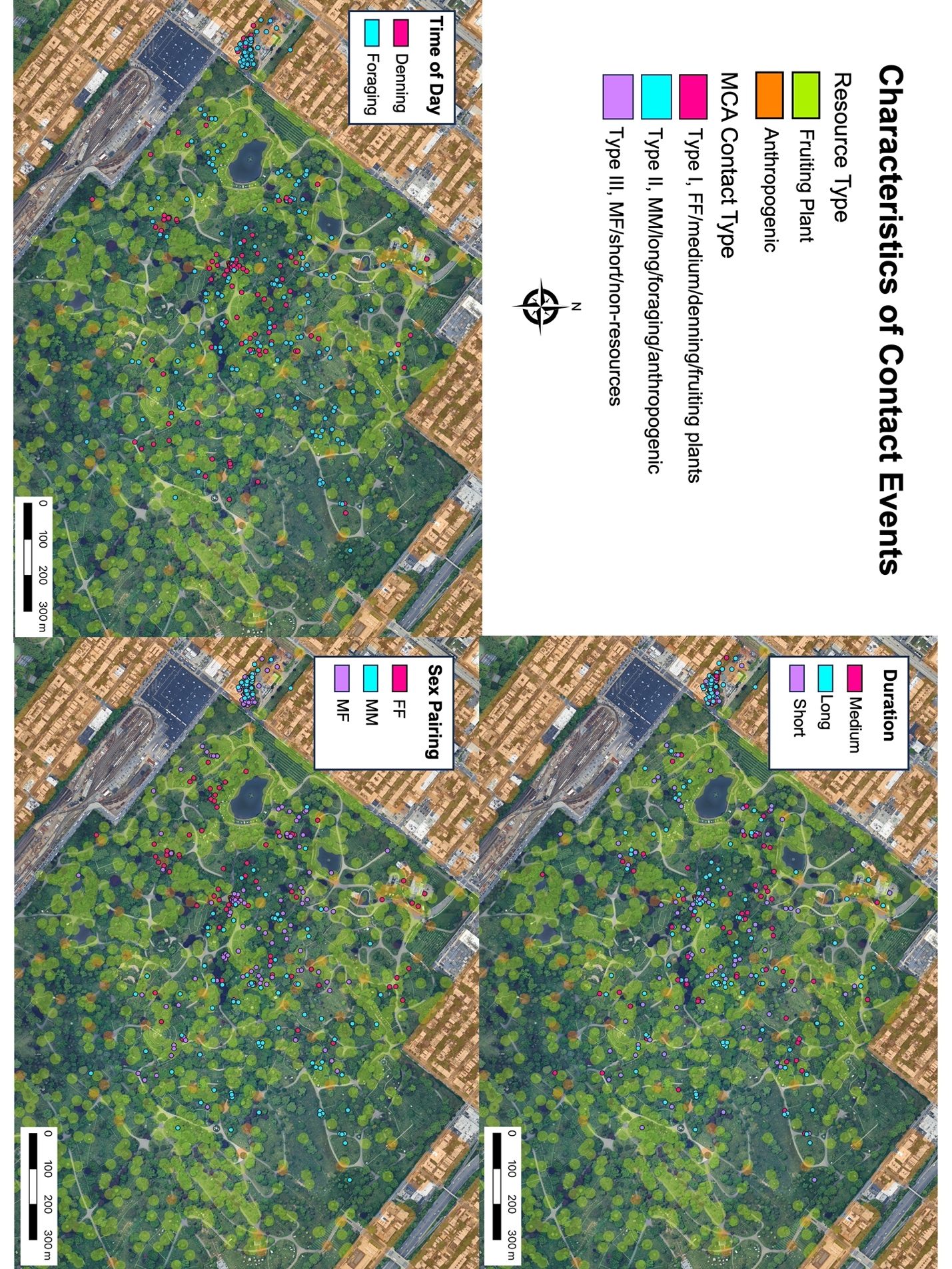


**Supplement, Figure 6***. Characteristics of* *366 contact events across the study site.* Contact events are overlaid on a satellite map of the study site with the presence of anthropogenic resources indicated by orange shading and the presence of fruiting plants indicated by green shading. Within each panel, contact events are colored by the level of each of the additional variables in the MCA analysis (contact duration, sex pairing, time of day) to which the contact event belongs. Across panels, levels of each of the variables that are associated with the same contact type are colored the same to indicate variable categories/contacts belonging to the same typology.
